# Development of an Oral Health Index and Its Association with Oral Health-Related Quality of Life and Cardiovascular Risks: A Cross-Sectional Study

**DOI:** 10.3390/ijerph23020195

**Published:** 2026-02-03

**Authors:** Vanessa Carvajal Soto, Larissa Knysak Ranthum, Luiz Felipe Manosso Guzzoni, Marcela Claudino, Eduardo Bauml Campagnoli, Marcelo Carlos Bortoluzzi

**Affiliations:** 1 Dentistry Post-Graduate Program, State University of Ponta Grossa (UEPG), Ponta Grossa 84030-900, Brazil; vanessacarvajalsoto@gmail.com (V.C.S.); lari_ranthum@hotmail.com (L.K.R.); 2 University Hospital, State University of Ponta Grossa (UEPG), Ponta Grossa 84030-900, Brazil; lfguzzoni@yahoo.com.br; 3 Health Sciences Post-Graduate Program, State University of Ponta Grossa (UEPG), Ponta Grossa 84030-900, Brazil; marcelaclaudino@hotmail.com (M.C.); ebcampagnoli@yahoo.com.br (E.B.C.)

**Keywords:** cardiovascular disease, oral health-related quality of life, tooth loss, periodontitis, oral diseases

## Abstract

**Highlights:**

**Public health relevance—How does this work relate to a public health issue?**

- Oral health diseases are highly prevalent worldwide and are increasingly recognized as contributors to systemic inflammation and cardiovascular risk.
- Current public health approaches often rely on fragmented oral indicators; this study addresses the need for a standardized, clinically feasible measure of overall oral health burden.

**Public health significance—Why is this work of significance to public health?**

- By demonstrating that poorer oral health is strongly associated with worse oral health-related quality of life and a greater burden of cardiovascular risk factors, this work reinforces the role of oral health as an integral component of general health.
- The development and validation of the Oral Health Index (OHI) provide a novel, objective tool that can strengthen epidemiological surveillance, interdisciplinary research, and health monitoring.

**Public health implications—What are the key implications or messages for practitioners, policy makers and/or researchers in public health?**

- Practitioners may use the OHI as a practical screening tool to identify individuals at increased systemic health vulnerability, supporting earlier prevention and integrated care strategies.
- Policymakers and researchers can incorporate the OHI into population-based programs and studies to better evaluate oral–systemic health interactions and inform evidence-based public health planning and resource allocation.

**Abstract:**

The OHI demonstrated moderate internal consistency and consistent associations with oral health-related quality of life and cardiovascular risk indicators. Objective: The primary objective was to propose and internally assess an Oral Health Index (OHI) which integrates multiple clinically assessed oral health variables. The secondary objective was to investigate its association with oral health-related quality of life (OHRQoL) and common clinical cardiovascular risk (CVR) factors. Material and Methods: This observational study included 191 participants. Seven parameters (tooth loss, periodontal disease, endodontic involvement, residual roots, extractions due to periodontitis, inflammatory oral mucosal diseases, and dental maintenance and rehabilitation status) were combined using Z-scores to compute the OHI, with higher scores indicating poorer oral health. CVR factors included age/sex thresholds, education level, BMI, smoking status, diabetes, hypertension, pulse pressure, and lung function. OHRQoL was assessed using the Oral Health Impact Profile. Results: Higher OHI scores were associated with poor oral health-related quality of life. Participants with cardiovascular risk factors had significantly higher OHI scores. The analysis demonstrated that the OHI was directly associated with worse oral health-related quality of life and a greater cardiovascular risk burden, independent of age, sex, and comorbidities. Conclusions: This study proposed and internally assessed the Oral Health Index, designed to integrate multiple clinical parameters into a single standardized measure of oral health. The OHI demonstrated moderate internal consistency and showed consistent associations with poorer oral health conditions, reduced oral health-related quality of life, and a greater cardiovascular risk burden.

## 1. Introduction

There is a well-established association between poor oral health, including periodontal disease and tooth loss, and an increased risk of cardiovascular diseases (CVDs) [1,2,3,4,5,6]. Several studies, including systematic reviews and meta-analyses consistently support this link, highlighting oral health as a potential modifiable risk factor for systemic conditions [1,2,6]. Among oral health indicators, tooth loss has been particularly associated with heightened risks of myocardial infarction, stroke, and cardiovascular mortality [4,6,7].

The biological mechanisms underpinning the relationship between oral and cardiovascular health are multifaceted, with chronic inflammation playing a central role [2,6,8]. Periodontal infections can trigger systemic inflammatory responses, leading to elevated levels of pro-inflammatory cytokines and other biomarkers [6,8]. Additionally, translocation of periodontal bacteria into the bloodstream may cause endothelial damage, vascular dysfunction, foam cell formation, and destabilization of atherosclerotic plaques, all of which contribute to arterial stiffness and atherogenesis [8,9]. Furthermore, tooth loss may impair masticatory function and negatively affect dietary habits and nutritional status, factors that can also influence cardiovascular outcomes [10]. Shared behavioral and socioeconomic risk factors, such as smoking, poor diet, obesity, and genetic predisposition, further complicate the interplay between oral and cardiovascular health [2,3,4,8].

A variety of oral health indicators have been investigated in relation to cardiovascular risk, including periodontal disease, tooth loss, endodontic involvement, residual roots, oral mucosal inflammatory conditions, and dental rehabilitation status [1,6,7,11,12]. Although these indicators provide valuable clinical information, they are most often examined in isolation, offering a fragmented view of oral health. Currently, there is a lack of clinically feasible composite indices capable of integrating multiple dimensions of oral health burden into a single standardized measure.

Therefore, the present study was not designed to re-establish the association between oral health and cardiovascular diseases, which is already well documented, but rather to address this methodological gap. The primary aim was to propose an Oral Health Index (OHI) that integrates seven clinically assessed parameters representing structural, inflammatory, and rehabilitative aspects of oral health. The secondary aim was to explore the association of this integrated oral health burden with oral health-related quality of life and a non-laboratory cardiovascular risk burden.

The present study addresses two main objectives. The primary aim is to develop and propose a comprehensive Oral Health Index (OHI) that consolidates seven clinically assessed parameters into a single, standardized score. The secondary aim is to evaluate the association between the OHI and both oral health-related quality of life and cardiovascular risk burden using non-invasive, clinically feasible methods. We hypothesize that higher OHI scores, indicating poorer oral health, will be significantly associated with greater cardiovascular risk and lower perceived oral health-related quality of life.

## 2. Materials and Methods

### 2.1. Study Design and Sample Characteristics

This study was classified as observational, cross-sectional, with prospectively collected data and exploratory in nature. It underwent review by the University Committee for Human Ethics Research and was approved under the number 5.942.632. Informed consent was obtained from all study participants. The study was conducted at the dental university clinics of the State University of Ponta Grossa (UEPG), located in Ponta Grossa, Paraná, Brazil.

The sample was obtained through convenience sampling, consisting of volunteers aged 18 years or older who were seeking consultation or dental treatment at the university clinics. Exclusion criteria included pregnancy, advanced mental illness or cognitive limitations that impaired the ability to understand the research procedures and questionnaires (illiterate participants were assisted), refusal to participate, and cases with missing sensitive data.

### 2.2. Sociodemographic and Medical Data

Sociodemographic and medical information, including gender, age, body mass index (BMI), education level, smoking status, previously diagnosed diseases, and medication usage, were initially collected through a structured anamnesis interview and clinical examination.

### 2.3. Risk Evaluation by the American Society of Anesthesiologists Classification (ASA)

A modified ASA classification system questionnaire for dentistry, the European Medical Risk Related History (EMRRH), was administered. The EMRRH is a patient-administered instrument originally developed and evaluated in a multicenter study conducted across several European countries to systematically document medical conditions and comorbidities relevant to dental care and to assess the associated level of medical risk [13].

### 2.4. Oral Health-Related Quality of Life Assessment

Oral health-related quality of life was assessed using the short-form Oral Health Impact Profile (OHIP-14). This questionnaire includes 14 items covering seven dimensions: functional limitation, physical pain, psychological discomfort, physical disability, psychological disability, social disability, and handicap. Responses were rated on a 5-point Likert scale (0 = never to 4 = very often). The total score ranged from 0 to 56, with higher scores indicating a greater negative impact.

### 2.5. Oral Health Condition Assessment

A comprehensive clinical oral examination was performed by two trained and calibrated licensed dentists enrolled in postgraduate training programs. Diagnostic uncertainties were resolved with three experienced professors of oral medicine, who were responsible for identifying chronic inflammatory conditions.

(1) Tooth Loss (TL): Calculated by subtracting the number of present teeth (excluding residual roots) from the total of 32 permanent teeth, including third molars [14].
(2) Dental Maintenance and Rehabilitation Status (DMRS): Classified per arch, from 0 (edentulous without prosthesis) to 6 (fully dentate). Total score ranged from 0–12 [14]. For index purposes, the score was inverted (inDMRS) to align higher values with worse oral health.
(3) Periodontal Disease (PerD): Diagnosed based on ≥2 sites with attachment loss ≥ 3 mm and ≥2 sites with probing depth ≥4 mm (not on the same tooth), or 1 site with PD ≥ 5 mm [15].
(4) Endodontic Involvement (EI): Number of teeth requiring endodontic treatment, based on clinical and radiographic criteria.
(5) Residual Roots (RR): Number of teeth with root fragments due to caries, indicating extraction.
(6) Exodontia due to Periodontitis (EP): Teeth indicated for extraction due to advanced periodontitis. Each tooth was assigned exclusively to one category (EI, RR, or EP).
(7) Inflammatory Disease of the Oral Mucosa (IDM): Presence of clinically detectable chronic inflammatory conditions, including denture-related stomatitis, traumatic ulcers, oral lichen planus, oral cancer, and osteoradionecrosis.

### 2.6. Development of the Oral Health Index (OHI)

The Oral Health Index (OHI) was developed as a composite measure to quantify the cumulative burden of oral diseases and the need for dental rehabilitation. The index includes seven clinically assessed parameters: tooth loss (TL), periodontal disease (PerD), endodontic involvement (EI), residual roots (RR), teeth indicated for extraction due to periodontitis (EP), inflammatory diseases of the oral mucosa (IDM), and the inverted dental maintenance and rehabilitation score (inDMRS). These variables were selected for their clinical and epidemiological relevance, representing structural, functional, and inflammatory dimensions of oral health. The index prioritizes cumulative and clinically consequential conditions rather than transient or behavior-dependent measures; dental caries experience, restorations, and oral hygiene indices were therefore not directly included, although their long-term consequences are indirectly captured through variables such as residual roots, endodontic involvement, tooth loss, extractions, and rehabilitation status.

To enable the integration of these variables into a single metric and ensure statistical comparability, all components were standardized using Z-scores, calculated as follows:

Z = (X − mean)/standard deviation (e.g., for number of teeth lost (TL) = TL − MEAN(TL))/SD(TL)(1)

The final OHI score was calculated as the arithmetic mean of the Z-scores of the seven parameters:

OHI = (Z_TL + Z_PerD + Z_EI + Z_RR + Z_EP + Z_IDM + Z_inDMRS)/7(2)

where:

TL = number of teeth lost;

PerD = presence of periodontal disease (dichotomous);

EI = number of teeth requiring endodontic treatment;

RR = number of non-restorable residual roots;

EP = number of teeth indicated for extraction due to periodontitis;

IDM = presence of chronic inflammatory diseases of the oral mucosa (dichotomous);

inDMRS = inverted score of dental maintenance and rehabilitation (sum of maxillary and mandibular scores; inDMRS = 12 − DMRS).

Higher OHI values indicate a worse overall oral health condition.

### 2.7. Validity and Reliability Analyses

To evaluate the internal psychometric properties of the Oral Health Index (OHI), different analytical approaches were applied. (1) Internal consistency was assessed using Cronbach’s alpha (α) and McDonald’s omega (ω), providing estimates of the coherence among the OHI components. (2) Construct validity was examined through correlation analyses between the OHI and each of its individual clinical components, testing whether the index appropriately reflected the multidimensional nature of oral health. (3) Convergent validity was evaluated by analyzing the association between OHI scores and oral health-related quality of life, as measured by the OHIP-14, under the expectation that poorer oral health would be linked to worse perceived quality of life. (4) Criterion-related (external) associations were examined by comparing OHI scores with established clinical cardiovascular risk (CVR) factors, both individually and as a total risk burden, to verify the index’s ability to reflect systemic health vulnerability.

### 2.8. Clinical Cardiovascular Risk Assessment

To investigate the potential association between oral health and cardiovascular vulnerability, a comprehensive clinical cardiovascular risk assessment was conducted using non-invasive, easily applicable measures suitable for dental settings. The assessment was based exclusively on observable clinical parameters and self-reported medical history, without the use of laboratory tests or blood analyses. Sociodemographic and clinical data were collected through a structured research questionnaire and physical examination. Key variables included age, sex, education level, body mass index (BMI), blood pressure, pulse pressure, lung function, smoking status, and the presence of chronic conditions such as diabetes and hypertension. Each variable was selected based on its well-established relevance to cardiovascular risk and classified according to recognized clinical guidelines and validated thresholds. The criteria applied to define individual cardiovascular risk indicators are described below:

(1) Age and Sex: Participants were classified as having an increased cardiovascular risk based on age and sex-specific thresholds, following established clinical guidelines. Men aged 45 years or older and women aged 55 years or older were considered at elevated risk for cardiovascular events. These thresholds reflect the typical onset age for increased cardiovascular vulnerability in each sex [16,17,18,19]. For cardiovascular risk assessment, this variable was scored dichotomously as either risk present or absent.
(2) Education Level: Lower educational attainment has been consistently associated with increased cardiovascular risk factors and adverse outcomes [20,21]. Educational level was obtained through a structured interview and categorized as: less than 9th grade, 9th to 11th grade or equivalent, and higher education. Following previous studies, participants with less than a high school education or equivalent were classified as having increased cardiovascular risk [20,21].
(3) Body Mass Index: Obesity, defined as a Body Mass Index (BMI) of 30 kg/m^2^ or higher, is widely recognized as an independent risk factor for cardiovascular disease [21,22,23]. For cardiovascular risk assessment, participants were classified dichotomously as either having obesity (present) or not having obesity (absent).
(4) Smoking Status: Smoking is a well-established cardiovascular risk factor, contributing significantly to the development and progression of cardiovascular diseases [16,19,21]. Smoking status was obtained through a structured research questionnaire and interview. For cardiovascular risk assessment, this variable was scored dichotomously as either risk present (smoker) or absent (non-smoker).
(5) Diabetes Mellitus: Diabetes mellitus is considered a major cardiovascular risk factor due to its association with vascular dysfunction and increased incidence of cardiovascular events [17,21]. Diabetes was defined by prior patient medical history. For cardiovascular risk assessment, this variable was scored dichotomously as either risk present (diabetic) or absent (non-diabetic).
(6) Hypertension: Hypertension was defined either by a prior medical diagnosis, prescribed medication and/or by consistently elevated blood pressure readings, with systolic blood pressure (SBP) ≥ 140 mm-Hg and/or diastolic blood pressure (DBP) ≥ 90 mm-Hg. Measurements were taken using a multiparameter cardiac monitor (Inmax12 Monitor, Instramed Co., Porto Alegre, Brazil) after a five-minute period of seated rest. The classification criteria were based on the 2017 Guideline for the Prevention, Detection, Evaluation, and Management of High Blood Pressure in Adults [24]. For cardiovascular risk assessment, this variable was scored dichotomously as either risk present (hypertensive) or absent (non-hypertensive).
(7) Pulse Pressure: Pulse pressure was calculated as the difference between systolic blood pressure (SBP) and diastolic blood pressure (DBP). Measurements were taken using a multiparameter cardiac monitor (Inmax12 Monitor, Instramed Co., Brazil) after a five-minute period of seated rest. A value of ≥60 mm-Hg was considered indicative of increased cardiovascular risk, in line with evidence linking widened pulse pressure to vascular aging and adverse cardiovascular outcomes [25].
(8) Lung function: Lung function was assessed via spirometry, following the technical standards [26]. Tests were conducted with participants in a standing position using a calibrated portable spirometer (Contec SP10, Contec Medical Systems Co., Qinhuangdao, China), and all measurements were performed by a single trained examiner to ensure consistency. The highest values of forced expiratory volume in one second (FEV1) and forced vital capacity (FVC) from acceptable and reproducible maneuvers were recorded. The FEV1/FVC ratio was calculated using the Omni Calculator platform (https://www.omnicalculator.com (accessed on 25 September 2025)), incorporating age, sex, height, and ethnicity. A ratio below 70% was considered indicative of airflow limitation and associated with increased cardiovascular risk [26].

For the purposes of analysis, cardiovascular risk (CVR) was evaluated in two ways: (1) as individual variables (present or absent), allowing for the assessment of specific associations between the Oral Health Index (OHI) and each cardiovascular risk factor independently; and (2) as a composite CVR score, calculated by summing the number of risk factors present for each participant. This cumulative score reflects the overall cardiovascular risk burden, with higher scores indicating a greater number of coexisting risk indicators. This dual approach enables both detailed and aggregate evaluation of the relationship between oral health status and cardiovascular risk.

### 2.9. Sample Size

The sample size was determined using an a priori power analysis conducted in JAMOVI (Power Analysis for Common Research Designs Module, Version 2.7.5, Sydney, Australia), considering the correlations between the Oral Health Index (OHI) and both the total sum of cardiovascular risk factors (CVR Total sum) and the OHIP-14 scores. Assuming an expected effect size (ρ) of 0.20, a statistical power of 80%, and a significance level (α) of 0.05, a minimum of 190 participants was required to reliably detect significant associations.

### 2.10. Data Analysis and Statistical Procedures

The JAMOVI software (JAMOVI project, Version 2.7.5, Sydney, Australia) were utilized for data analysis, employing descriptive and inferential methods. Two-tailed significance level of *p* ≤ 0.05 was considered statistically significant. The assumptions for data normality where verified through Shapiro–Wilk, Kolmogorov–Smirnov and Anderson-Darling. Correlation tests with non-normal data distributions were analyzed through Spearman Correlation test. Mann–Whitney test was used to compare the differences between two independent samples.

**Figure 1:**
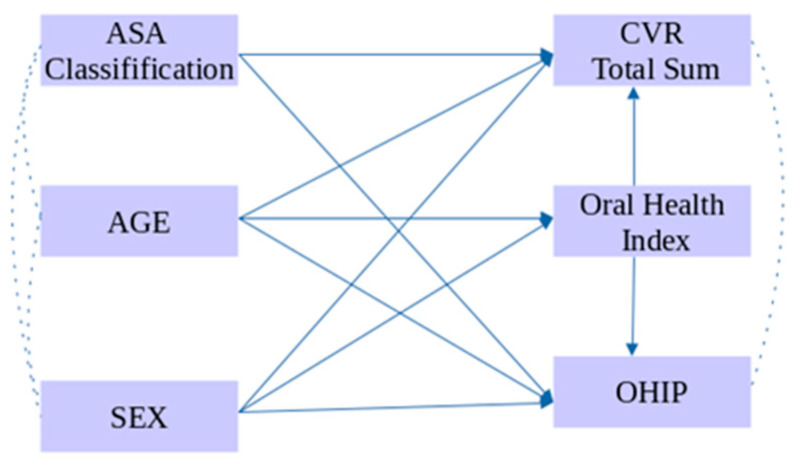
Conceptual GLPA model illustrating proposed relationships between sociodemographic variables, systemic health, oral health (OHI), quality of life (OHIP-14), and cardiovascular risk (CVR). Analyses tested direct/indirect effects.

A generalized linear path analysis (GLPA) model was employed to examine the interrelationships among variables and their associations with cardiovascular risk (sum of the risks), OHIP-14, and the Oral Health Index, incorporating sex, age, and ASA classification (healthy versus presence of any comorbidity). Model validity was assessed using the following fit indices: χ^2^ (Chi-square), SRMR (Standardized Root Mean Square Residual), RMSEA (Root Mean Square Error of Approximation), CFI (Comparative Fit Index), GFI (Goodness-of-Fit Index), and adjusted GFI (Adjusted Goodness-of-Fit Index). GLPA is a robust statistical method that enables simultaneous examination of multiple variable influences, identifying both direct and indirect effects. This approach facilitates a more precise and comprehensive interpretation of complex relationships within clinical data. The conceptual hypothesis of the GLPA is illustrated in Figure 1.

## 3. Results

The study sample comprised 191 patients, with ages ranging from 19 to 86 years (mean ± SD: 45 ± 16 years), as summarized in Table 1. The majority of participants were female (*n* = 130; 68%). With respect to educational attainment, 22.0% had completed high school, and 20.4% had some college education without graduation. Additionally, 20.4% had not completed elementary school. Smaller proportions of the sample had incomplete high school education (14.7%) or had completed elementary education (7.9%). Only 6.8% had attained higher education, and 6.3% held a postgraduate degree. A minority of participants (1.6%) were illiterate.

### 3.1. Oral Health Conditions

The patient sample exhibited a mean of 12 missing teeth, with the number ranging from zero to complete edentulism. Among the participants, 8% were fully edentulous, 29% had fewer than 12 remaining teeth, and 38% presented with 20 or fewer teeth. Information on dental rehabilitation status is summarized in Table 2. Periodontal disease was diagnosed in 31% of the sample, whereas 13% had at least one tooth indicated for extraction as a result of periodontal disease (EP). Furthermore, 7% presented with at least one tooth indicated for extraction due to severe carious destruction (residual root—RR), and 8% had at least one tooth requiring endodontic treatment (EI). Inflammatory conditions of the oral mucosa were identified in 53 patients, with prosthesis-related lesions being the most prevalent, observed in 32 cases (60%).

### 3.2. Oral Health Index (OHI)

The Oral Health Index (OHI) exhibited a mean value approximating zero. Individual scores ranged from −0.595 to 2.08, with a standard deviation of 0.528, reflecting substantial variability in oral health conditions within the sample. The mode was −0.539, indicating that the most frequently observed scores corresponded to better oral health status relative to the sample mean.

The OHI showed strong and significant correlations with its individual clinical components, indicating internal coherence among the selected oral health parameters. The highest correlations were observed with tooth loss (ρ = 0.83, *p* < 0.001) and the inverted Dental Maintenance and Rehabilitation Status (inDMRS) (ρ = 0.81, *p* < 0.001). Moderate correlations were found with inflammatory disease of the oral mucosa (ρ = 0.53, *p* < 0.001), periodontal disease (ρ = 0.52, *p* < 0.001), and the need of exodontia due to periodontitis (ρ = 0.43, *p* < 0.001). Weaker but still significant correlations were identified with endodontic involvement (ρ = 0.26, *p* < 0.001) and residual roots (ρ = 0.26, *p* < 0.001). Together, these associations demonstrate that the OHI consistently reflects the combined contribution of multiple oral health conditions.

The index also demonstrated moderate internal consistency, with a Cronbach’s alpha of 0.57 and a McDonald’s omega of 0.64. These values reflect the multidimensional nature of the index, indicating that while the included oral health components are related, they also capture distinct aspects of oral health burden.

Table 3 presents the relationship between various oral health clinical conditions and the Oral Health Index (OHI). The results demonstrate that participants with poorer oral health conditions consistently exhibited higher (positive) OHI scores, whereas those with better oral health had negative mean OHI values. These findings confirm that lower (negative) OHI values are associated with better oral health status, supporting the index’s ability to differentiate oral health burden effectively.

### 3.3. Oral Health Index (OHI) and Oral Health-Related Quality of Life (OHIP-14)

The Oral Health Index (OHI) was significantly correlated with all dimensions of the OHIP-14, supporting its convergent validity. The strongest association was observed with psychological disability (Spearman’s rho = 0.40, *p* < 0.001), followed by the overall OHIP total score (Spearman’s rho = 0.38, *p* < 0.001). Moderate correlations were also found with functional limitation (Spearman’s rho = 0.34, *p* < 0.001), physical disability (Spearman’s rho = 0.29, *p* < 0.001), and handicap (Spearman’s rho = 0.29, *p* < 0.001). Weaker but still significant correlations were seen with social disability (Spearman’s rho = 0.24, *p* < 0.001), physical pain (Spearman’s rho = 0.20, *p* = 0.004), and psychological discomfort (Spearman’s rho = 0.19, *p* = 0.008). These results indicate that poorer oral health, as reflected by higher OHI scores, is consistently associated with greater negative impacts on oral health-related quality of life.

### 3.4. General Health Condition, Cardiovascular Risk and Oral Health Index (OHI)

Table 4 shows the association between the Oral Health Index (OHI) and various clinical cardiovascular risk (CVR) factors. Participants classified as having cardiovascular risk consistently presented significantly higher mean OHI scores, indicating poorer oral health status, with the exception of lung function (observed FEV1/FVC ratio), which did not show a significant difference. Additionally, it was observed a strong correlation between the OHI and the total number of cardiovascular risk factors (Spearman’s rho = 0.65, *p* < 0.001), which remained significant even after controlling for age and sex (partial Spearman’s rho = 0.29, *p* < 0.001), showing that the association is not solely explained by these confounding variables. These consistent associations across multiple risk factors support the criterion-related (external) validity of the OHI, suggesting its potential utility as an indicator of systemic health vulnerability and further reinforcing the link between oral and systemic health.

### 3.5. Interactions Among the Variables

The GLPA included all 191 observations, with 17 free parameters and 3 endogenous variables (OHIP-14, OHI and the total sum of CVR). The model demonstrated validity, as indicated by the following fit indices: χ^2^ (*p* = 0.41), SRMR = 0.008, RMSEA = 0.00 (*p* = 0.51), CFI = 1.0, GFI = 0.990, and adj. GFI = 0.98. Table 5 presents the results of the structural equation modeling (SEM) examining the relationships among the Oral Health Index (OHI), oral health-related quality of life (OHIP Total), and cardiovascular risk (CVR Total sum), while controlling for age, sex, and comorbidities (ASA classification). The analysis confirmed that poorer oral health (OHI) was associated with worse quality of life and a greater cardiovascular risk burden, independent of age, sex, and comorbidities. These findings also reinforce the criterion-related (external) and convergent validity of the OHI, highlighting its value in capturing the interplay between oral health, perceived quality of life, and cardiovascular risk.

## 4. Discussion

The proposed Oral Health Index (OHI) was developed to provide a standardized measure of overall oral health burden by integrating multiple clinically assessed parameters. Traditional oral health indicators, such as tooth loss and periodontal status, often offer a fragmented view and may not fully capture the cumulative burden of oral diseases or the extent of dental rehabilitation needs. By incorporating seven distinct variables, including both disease-specific (e.g., periodontitis, endodontic involvement, residual roots, inflammatory disease of oral mucosa) and rehabilitation-related components (e.g., dental maintenance and prosthetic status), the OHI allows for a more holistic assessment of oral health. The OHI integrates several key clinical parameters that are directly or indirectly associated with chronic inflammation in the oral cavity; therefore, a higher OHI score, representing poorer oral health, could be interpreted as indicating a greater potential oral inflammatory burden, which may contribute to systemic inflammation and help explain observed links with cardiovascular risk.

The results demonstrated significant correlations between each clinical component and the OHI, indicating internal coherence among the selected oral health parameters. Higher OHI scores were consistently associated with poorer clinical oral conditions, supporting the interpretation of the index as a multidimensional measure of oral health burden rather than a reflection of a single condition. These findings suggest that the OHI captures the combined contribution of structural damage, inflammatory conditions, and rehabilitation needs within the studied sample.

These findings are consistent with previous epidemiological and cohort studies demonstrating that cumulative oral health deterioration, particularly tooth loss and periodontal disease, is associated with increased cardiovascular risk and poorer health outcomes. Prior studies have shown that tooth loss and severe periodontal disease are linked to hypertension, stroke, atherosclerosis, and cardiovascular mortality. The present results extend this evidence by demonstrating that an integrated oral health burden, captured through a composite index, is strongly associated with both cardiovascular risk indicators and oral health-related quality of life.

A consistent and well-established association exists between oral health conditions, particularly periodontal disease (gingivitis and periodontitis), and tooth loss, and an increased risk of cardiovascular disease and other systemic health issues [1,2,3,4,5,6,8,27]. This link encompasses a range of cardiovascular outcomes, including coronary heart disease, myocardial infarction, stroke, hypertension, atherosclerotic cardiovascular disease, arterial stiffness, carotid artery calcification, and overall increased cardiac death and all-cause mortality [1,2,4,5,6,8,9,10]. For instance, individuals with significant tooth loss have been found to have a higher risk of stroke [4] and hypertension [10], and patients with severe periodontal disease show increased odds of developing cardiovascular diseases [8]. Even in populations with low traditional risk factors for cardiovascular disease, damaged teeth with exposed pulp were associated with higher inflammation and aortic valve calcification [6].

The primary mechanisms underlying this association involve chronic inflammation and the systemic dissemination of oral bacteria [1,4,6,8,12]. Inflammation in periodontal tissues can contribute to a systemic inflammatory response, marked by elevated C-reactive protein (CRP) and pro-inflammatory cytokines, which may lead to endothelial dysfunction, increased atherosclerosis, and a pro-thrombotic state [1,2,3,6,8,10,12]. Furthermore, tooth loss often reflects a history of poor oral hygiene and can lead to altered dietary habits and nutritional deficiencies, while sharing common risk factors like smoking and obesity, all of which contribute to an elevated cardiovascular risk [8,12,27]. Beyond cardiovascular health, poor oral health and tooth loss are also consistently associated with an elevated risk of other systemic conditions, including diabetes, lower limb complications, kidney complications, and cognitive decline, including dementia [1,6,8,11,12,27,28]. These findings highlight the potential public health relevance of integrating oral health into broader strategies for disease prevention and management [1,2,12].

In this context, the findings of the present study strengthen the biological plausibility of the proposed OHI as a systemic health marker. Higher OHI scores, reflecting a greater burden of periodontal disease, tooth loss, endodontic involvement, residual roots, inflammatory mucosal disease, and inadequate rehabilitation, were consistently associated with a higher prevalence of clinical cardiovascular risk factors and with reduced oral health-related quality of life. These associations mirror the mechanisms previously described, whereby cumulative oral inflammation and infection contribute to systemic inflammatory responses, endothelial dysfunction, and atherosclerosis. Thus, the OHI not only captures the multidimensional nature of oral health deterioration but also provides a composite measure that aligns with the pathways linking oral conditions to cardiovascular vulnerability, reinforcing its potential role as a clinically feasible indicator for exploring oral–systemic health relationships.

### Study Limitations

This study has some limitations. Its cross-sectional design precludes causal inference between oral health and cardiovascular risk. Although the OHI demonstrated consistent internal coherence and meaningful associations within the studied sample, further evaluation in independent, larger, and diverse populations is required to confirm its generalizability. Cardiovascular risk factors were clinically assessed using pragmatic, non-invasive measures suited to dental settings, but no biochemical markers (e.g., CRP, lipid profile) were included, which might underestimate the true risk profile. Additionally, residual confounding by lifestyle factors such as diet, physical activity, and socioeconomic determinants cannot be fully excluded. Although examinations were performed by trained examiners with expert supervision, formal calibration procedures and inter-/intra-examiner reliability metrics were not assessed, which may have introduced measurement variability. The findings of this study should be interpreted as supportive evidence of the external relevance and applicability of the OHI, not as definitive validation of the instrument. Full validation of the OHI will require replication in independent samples, longitudinal designs, and, ideally, comparison with alternative composite oral health measures when available.

## 5. Conclusions

This study proposed an Oral Health Index (OHI) designed to integrate selected clinically assessed parameters into a single standardized measure of oral health burden. The OHI demonstrated consistent internal coherence and meaningful associations with oral health-related quality of life and cardiovascular risk indicators. While the index captures important structural, inflammatory, and rehabilitative dimensions of oral health, it does not encompass all possible determinants, such as dental caries experience, restorative status, or oral hygiene behaviors. Therefore, the OHI should be interpreted as a clinically feasible indicator of cumulative oral health burden, with potential for future refinement and external validation in diverse populations.

## Figures and Tables

**Table 1:** Demographic characteristics of the study participants (*n* = 191).

Variable	Category	*n*	%
Age (years)	Mean ± SD	–	45 ± 16
	Range	–	19–86
Sex	Female	130	68.0
	Male	61	32.0
Educational level	Illiterate	3	1.6
	Incomplete elementary school	39	20.4
	Completed elementary school	15	7.9
	Incomplete high school	28	14.7
	Completed high school	42	22.0
	Some college (no degree)	39	20.4
	Higher education	13	6.8
	Postgraduate degree	12	6.3

– indicates that the value is not applicable, as age range does not correspond to a frequency count.

**Table 2:** Distribution of Dental Maintenance and Rehabilitation Status (DMRS) Scores for Maxilla and Mandible.

Mandible		Maxilla		Oral Function Status and Rehabilitation Index	
%	*n*	%	*n*		Score
4.2	8	2.6	5	Fully edentulous arch without prosthetic rehabilitation (The patient does not possess or does not use a complete prosthesis in the arch)	0
6.3	12	19.9	38	Fully edentulous arch with complete prosthetic rehabilitation (The individual uses a complete removable prosthesis in the arch)	1
20.4	39	8.4	16	Partially edentulous arch without prosthetic rehabilitation (The arch is missing some teeth and no prosthetic replacement is used)	2
12.0	23	10.5	20	Partially edentulous arch with removable partial prosthesis (The arch are replaced with a removable prosthetic device, but not all teeth were replaced)	3
1.6	3	2.6	5	Partially or totally edentulous arch with fixed prosthetic rehabilitation or implants (The arch is restored using fixed bridges or implant-supported restorations—including fixed full-arch prostheses. There may still be some missing teeth or not all teeth were functionally replaced)	4
17.8	34	16.8	32	Functional dentition preserved in the arch through the presence of most natural teeth, or complemented with fixed prostheses, or implants.	5
37.7	72	39.3	75	Functional dentition preserved in the arch with most natural teeth present	6

**Table 3:** Association Between the Oral Health Index (OHI) and Clinical Oral Health Conditions.

*p*-Value ^a^	OHI (Mean)	Parameter	Oral Health Clinical Conditions
<0.001	0.46	20 teeth or less	Amount of Teeth
	−0.27	More than 20 teeth	
<0.001	0.43	Present	Periodontal Disease
	−0.19	Absent	
<0.001	0.50	At least 1 tooth with EI	Endodontic Involvement (EI)
	−0.04	None teeth with EI	
<0.001	0.77	At least 1 tooth with RR	Residual Roots (RR)
	−0.05	None residual root	
<0.001	0.64	At least 1 tooth with EP	Exodontia due Periodontitis (EP)
	−0.09	None teeth with EP	
<0.001	0.42	Yes	Inflammatory Disease of the Oral Mucosa (IDM)
	−0.37	No	
<0.001	0.43	Poorly Rehabilitated (7 points or more)	Inverted Dental Maintenance and Rehabilitation Status (inDMRS)
	−0.24	Moderately Rehabilitated (Up to 6 points)	

^a^: Based in the Mann–Whitney test.

**Table 4:** Association Between the Oral Health Index (OHI) and Clinical Cardiovascular Risk Factors.

Mann–Whitney (OHI-CVR)	OHI (Mean) CVR-Yes	OHI (Mean) CVR-No	CVR-Yes (%)	CVR-No (%)	Variables for Clinical Cardiovascular Risk
<0.001	0.28	−0.22	84 (44)	107 (56)	Age/Gender (Males: ≥45 years/Females: ≥55 years)
<0.001	0.31	−0.25	85 (44.5)	106 (55.5)	Educational level (Below high school/High school or above)
<0.001	0.25	−0.16	46 (24.1)	145 (75.9)	Body Mass Index (BMI) (≥30)
<0.001	0.24	−0.07	42 (22)	149 (78)	Smoker (no/yes)
<0.001	0.40	−0.06	25 (13.1)	166 (86.9)	Diabetes (no/yes)
<0.001	0.16	−0.19	55 (28.8)	136 (71.2)	Hypertension (yes: systolic 140–159 mm-Hg and or diastolic 90–99 mm-Hg)
<0.001	0.19	−0.05	39 (20.4)	152 (79.6)	Pulse Pressure (no risk < 60/CV risk yes ≥ 60)
NS	−0.03	9	40 (20.9)	151 (79.1)	Observed FEV1/FVC ratio (no risk ≥ 70%/CV risk yes < 70%)
<0.001	0.10	−0.54	159 (83.2)	32 (16.8)	Total Sum of Cardiovascular Risks

**Table 5:** Structural equation modeling of the relationships among Oral Health Index (OHI), oral health-related quality of life (OHIP-14), and cardiovascular risk, adjusted for age, sex, and comorbidities.

*p*-Value	β	Endogenous (Dependent Variable)	Exogenous Variables
<0.001	0.34	OHIP Total	OHI
0.002	−0.20	OHIP Total	Sex
0.29	−0.09	OHIP Total	Age
0.01	−0.17	OHIP Total	ASA (Comorbidity)
0.95	−0.003	OHI	Sex
<0.001	0.67	OHI	Age
0.02	0.12	CVR Total sum	Sex
<0.001	0.24	CVR Total sum	OHI
<0.001	0.41	CVR Total sum	Age
0.005	−0.16	CVR Total sum	ASA (Comorbidity)

## Data Availability

The dataset used for the current study is available from the corresponding author upon reasonable request.
